# Lessons learned in translating pain knowledge into practice

**DOI:** 10.1097/PR9.0000000000001100

**Published:** 2023-11-02

**Authors:** Juliane Becker, Philip R. Effraim, Sulayman Dib-Hajj, Heike L. Rittner

**Affiliations:** a Department of Anesthesiology, Intensive Care, Emergency and Pain Medicine, Center for Interdisciplinary Pain Medicine, University Hospital Würzburg, Würzburg, Germany; b Department of Anesthesiology, Yale School of Medicine, New Haven, CT, USA; c Department of Neurology, Center for Neuroscience & Regeneration Research, Yale School of Medicine, New Haven, CT, USA; d Rehabilitation Research Center, VA Connecticut Healthcare System, West Haven, CT, USA

**Keywords:** Sodium channel, Translational pain medicine, NGF, NK1R

## Abstract

**Introduction:**

During the past 2 decades, basic research deciphering the underlying mechanisms of nociception and chronic pain was thought to finally step beyond opioids and nonsteroidals and provide patients with new analgesics. But apart from calcitonin gene–related peptide antagonists, nothing arrived in hands of clinicians.

**Objectives:**

To present existing evidence of 3 representative target molecules in the development of novel pain treatment that, so far, did not result in approved drugs.

**Methods:**

This Clinical Update aligns with the 2022 IASP Global Year Translating Pain Knowledge into Practice and selectively reviews best available evidence and practice.

**Results:**

We highlight 3 targets: a ion channel, a neuronal growth factor, and a neuropeptide to explore why these drug targets have been dropped in clinical phase II–III trials. Antibodies to nerve growth factor had very good effects in musculoskeletal pain but resulted into more patients requiring joint replacements. Blockers of NaV1.7 were often not effective enough—at least if patients were not stratified. Blockers of neurokinin receptor were similarly not successful enough. In general, failure was most often to the result of a lack of effect and to a lesser extend because of unexpected severe side effects. However, all studies and trials lead to an enormous move in the scientific community to better preclinical models and testing as well as revised methods to molecularly phenotype and stratify patients.

**Conclusion:**

All stakeholders in the process can help in the future: better preclinical studies, phenotyping and stratifying patients, and participation in clinical trials to move the discovery of analgesics forward.

## Abstract 2

Using 3 molecular targets, the article explores the lack of translation of pain knowledge into the patient treatment and provides strategies to overcome these challenges.

## 1. Introduction

Molecular pain research is a relatively young field of a little more than 2 decades. Its beginning can be dated around 1995 with the discovery of P2X3 and Nav1.8 as genes enriched in dorsal root ganglion sensory neurons, partly expressing nociceptor-associated markers.^17,52^ Two further milestones to be mentioned are the discovery of the vanilloid receptors for noxious heat stimuli in 1997^16^ and the piezo receptors for mechanical stimulation in 2010^18^—both receiving the Nobel Prize in Medicine in 2021. The 2 decades have seen an explosive growth of investigations into the molecular architecture of primary afferents, the first neuronal link in the pathway that culminates in pain perception in the central nervous system (CNS). In parallel, biopsychosocial aspects of chronic pain have gained a scientific basis. These developments have advanced our understanding of the pain experience and identified new targets for the development of treatments for pain.

Chronic pain arises from many different pathological conditions. These include primary chronic pain diseases like nonspecific back pain and fibromyalgia or secondary chronic pain as a sequela of cancer, an injury or damage to the somatosensory systems resulting in neuropathic pain. Diagnosis and treatment have remained a challenge. Opioids and nonopioids such as nonsteroidal anti-inflammatory drugs and paracetamol as well as selected antineuropathic drugs dominate daily routine in the clinic. However, they often present with limited effectiveness and considerable side effects, such as increased risk of falls and addiction for opioids and gastrointestinal bleeding and increased cardiovascular events for nonopioids.

There has been an intense effort by basic science researchers, clinicians, and pharmaceutical industry to understand pain at the molecular level and to identify new targets for drug development. This effort has faced multiple challenges. Beside the clinical trial design, that is not only an expensive but also a time-consuming process, one must consider different issues including—amongst others—careful section of patients, (active) placebos as controls, proper randomization, sufficient duration if patients with chronic pain are treated, and enough power to detect and effect and objective biomarkers when they become available.^8^ In addition, in the absence of validated biomarkers, measuring pain in humans is far from objective, too. In fact, the widely used pain ratings are strongly affected by social, cognitive, and emotional factors, such as suffering, social desirability, context, and expectations.

Another important hurdle in pain research is that although laboratory animals are sentient, they cannot directly communicate their pain “perception” in a language to be understood easily by humans. So, basic science researchers must refer to “nociceptive behavior” or “pain-related behavior,” but this can also be highly affected by other variables, apparent or not well understood, which are not always taken into consideration. In addition, most of the models employed do not mirror the major clinical diseases. Pain is normally caused by impulses generated in the periphery, reaching a conscious brain. Although any treatment or drug may reduce paw withdrawal in a rodent animal model, this reflex response may not reflect a change in pain perception. This latter mechanism is hard to evaluate, although new and more sophisticated assays of affective components of pain are now expected by the research community and funding agencies. Finally, mice and men are different in the pain processing system, so extrapolation of preclinical data always carries risks, and the use of larger animal models and human tissues in these preclinical studies will hopefully bridge this knowledge gap.

Despite these limitations, some drugs have made it into the clinic including several anticonvulsants like gabapentin and pregabalin for neuropathic pain, albeit with limited efficiency, and antagonists or antibodies against calcitonin gene–related peptides (CGRP) for migraine.^29^ To date, 2 different classes targeting CGRP have been developed for migraine therapy: There are small molecule receptor antagonists of the CGRP receptor and blocking monoclonal antibodies. Their discovery started with CGRP measured in the blood of patients with migraine—so not entirely a bench to bedside. However, animal studies have confirmed the linkage of CGRP and migraine. Although the CGRP receptor is widely expressed, not only in the vascular tissues but also in the peripheral and central nervous system, no severe adverse effects, except elevation in transaminases in some subjects, have been reported to date. Therefore, CGRP receptor antagonists are a very effective and promising drug especially in migraine therapy.^21^ Other promising targets have not reached the clinic. Here, we will explore 3 drug targets and the lessons that they teach us. Out of the large group of molecules, we focused on 3 prominent ones from the group of growth factors, ion channels, and neuropeptides as targets—because they are highly relevant in the clinic.

## 2. Growth factors: nerve growth factor for the treatment of osteoarthritis and back pain

### 2.1. Nerve growth factor function and involvement in disease

Nerve growth factor was discovered in the 1950s as a tumor tissue produced soluble factor influencing the somatosensory system.^13^ Nerve growth factor belongs to the group of neurotrophins containing 3 subunits, called α, β, and γ.^58^ During the embryonic development of the peripheral nervous system, nerve growth factor (NGF) directs differentiation of sensory and sympathetic neurons from the neuronal crest. In adulthood, NGF maintains skeletal muscle function and immune homeostasis for splenocytes. Nerve growth factor binds to 2 separate cell surface receptors: p75 and tropomyosin receptor kinase A (TrkA). Tropomyosin receptor kinase A has a high affinity for NGF, whereas p75 might function more as a coreceptor. When NGF binds to TrkA, the receptor dimerises and is phosphorylated and therefore results in the activation of the intracellular protein kinase. This results not only in an induced gene transcription of substance P (SP) and calcitonin gene–related peptide but also in an increased translocation of transient receptor potential vanilloid 1 (TRPV1) channel proteins in the cell surface membrane. Because of a positive feedback loop in the NGF signaling, this results in an increased NGF-induced hyperalgesia.^40^ Substance P and CGRP elicit vasodilation and chemotaxis, causing subsequent neurogenic inflammation. Second, binding of NGF to TrkA sensitized nociceptors for the activation of acid-sensing ion channels or transient receptor potential vanilloid TRPV1.

### 2.2. Clinical studies and challenges

After the initial discovery of NGF and its proalgesic properties in preclinical models,^57^ the human equivalent was cloned, and selective NGF monoclonal antibodies were generated.^1^ They interfere with the functioning of TrkA-positive nociceptors and reduce their sensitivity to noxious stimuli.^37^ The first clinical osteoarthritis trial with tanezumab was conducted in 2010.^41^ Initial phase II and phase III clinical trials with NGF inhibitors were very promising and demonstrated efficacy not only in reducing joint pain but also in improving function.^49^ However, reports about a dose-dependent rapidly progressive osteoarthritis (RPOA) soon emerged. Rapidly progressive osteoarthritis is characterized by pain, rapid joint space narrowing, and severe atrophic bone changes with collapse of the subchondral area within one year ultimately requiring joint replacement. Observation of this side effect led to a clinical hold by the FDA between 2010 and 2015.^50^ Apparently, higher doses or a simultaneous application of non-steroidal anti-inflammatory drugs contributed to this side effect.^58^ Despite numerous efforts in subsequent analyses, it was not possible to predict by clinical, radiologic, or molecular biomarkers, which patients were at risk. Although there is no benefit for pain therapy in humans to be reported up to date, anti-NGF is approved by the European Medicines Agency for the treatment of painful arthritis in dogs (Librela|EuropeanMedicinesAgency(europa.eu)). So far, there are no reports about RPOA in this group of animals. Therefore, although science did not have any benefit for humans, this example shows how at least animal health can benefit from pain research.^27^

Unfortunately, analgesic effectiveness and RPOA are both dose dependent. Possible explanations for RPOA suggest abnormal joint loading either due to the sensory loss to feel pain or due to analgesic effects to reduce joint pain.^58^ Good analgesia but joint destruction could be back translated to the preclinical models: anti-NGF treatment alters the microenvironment including innervation and blood flow within the joint leading to accelerated joint degeneration because NGF is expressed in endothelial cells in subchondral bone layers in close proximity of TrkA-positive sensory fibers. Indeed, it is known that nociceptors regulate blood flow, and mice that lack TrkA have reduced bone formation.

### 2.3. Summary and conclusions

In summary, NGF antibodies are the classical molecular pathway that showed good analgesic effectiveness, but the unexpected side effects—albeit not common but severe—finally prohibited its use. There are several explanations focusing on the development of RPOA. Beside the molecular mechanism, trial patients were also considered to overdo because of less pain sensation. Because reducing the dose led to a reduction in RPOA, it is to be assumed that unsuspected pathways led to these unwanted side effects. Reducing the dose of anti-NGF significantly reduced the incidence of RPOA, but it also showed significantly less effectiveness in pain therapy. In the future, this can only be avoided if systematic screenings are employed in preclinical models to detect surprising pathways. Nevertheless, studies are still ongoing for the treatment of other painful diseases in which RPOA is important, such as eg, schwannomatosis (Clinical Trials No NCT04163419) or bone cancer pain (eg, Clinical Trials No NCT02609828).

## 3. Ion channels: NaV1.7 blocker for the treatment of neuropathic pain

### 3.1. Nav1.7 function and involvement in disease

Voltage-gated sodium (Nav) channel Nav 1.7, which is abundant in peripheral sensory and sympathetic neurons, plays a critical role in action potential formation and has been genetically and functionally validated as causing human pain disorders.^9,25^ Fully penetrant gain-of-function mutations in Nav1.7 cause the human painful disorders like inherited erythromelalgia (IEM) and paroxysmal episodic pain disorder (PEPD). Less penetrant mutations have been identified in idiopathic adult-onset small fiber neuropathy and in patients with diabetic painful neuropathy.^9^ By contrast, loss-of-function mutations in Nav1.7 cause Nav1.7-associated congenital indifference to pain (CIP) in which patients have a profound insensitivity to pain without any other apparent neurological abnormalities except anosmia.^9,25^ Nav1.7 has also been implicated in acquired pain after injury, inflammation, and diabetes.^24^ This has made this channel a prime target for the development of novel therapies for the treatment of pain.

### 3.2. Clinical studies and challenges of Nav1.7 blockers

There have been numerous clinical studies involving inhibitors of the Nav1.7 channel.^3^ Perhaps, the most extensively studied of these compounds are BIIB074 (also called vixotrigine) from Convergence/Biogen and PF-05089771 from Pfizer.

Vixotrigine is a state-dependent and use-dependent prolinamide blocker of Nav1.7, and thus, it is predicted to preferentially inhibit high firing neurons. However, it has now been shown not to be Nav1.7-selective.^22,35^ Vixotrigine received orphan drug status in 2013 and has been studied in 3 phase II clinical trials. A randomized, double-blind, placebo-controlled, crossover study that investigated the efficacy of vixotrigine in the treatment of neuropathic pain from lumbosacral radiculopathy in 82 subject^19^ met the primary end point and significantly reduced daily neuropathic pain compared with baseline (*P* = 0.0265). In another trial (2015),^6^ Biogen also stated that the drug was more effective in individuals who were not on concomitant pain medications at the time of the study—they reported a more significant reduction in their pain compared with baseline.^33^ However, another 502-person, 14-week study on patients with lumbosacral radiculopathy^56^ did not meet its primary or secondary end points. The third phase II trial^5^ was a multicenter, randomized, double-blind, placebo-controlled withdrawal trial for trigeminal neuralgia.^59^ The primary end point was treatment failure (defined as a 50% increase in either frequency of paroxysms or the intensity of pain during paroxysms). Although participants receiving vixotrigine experienced less treatment failure than placebo, 33.3% vs 64.3%, respectively, the result did not reach statistical significance. However, the secondary end points were met: vixotrigine reduced the number and severity of daily painful paroxysms, and the mean daily pain score compared with placebo.^59^ Biogen is no longer pursuing lumbosacral radiculopathy pain as an indication for vixotrigine; however will it continue to investigate it in trigeminal neuralgia^5^ and has completed a phase II trial to treat small fiber neuropathy, but no results have been posted to date.^26^

The first-in-class arylsulfonamide PF-05089771 compound from Pfizer is a potent, selective, and peripherally restricted Nav1.7 antagonist.^2^ A small proof-of-principle, double-blind, crossover, clinical study of PF-05089771 was conducted on 5 individuals with IEM. In preclinical studies using patient-specific sensory neurons differentiated from induced pluripotent stem cells (iPSC-SN), PF-05089771 reduced excitability of neurons from 3 of the 4 participants, suggesting potential efficacy in treating these patients.^14^ In the clinical study, participants received either a single oral dose of 1600 mg of PF-05089771 or placebo over 2 study periods, and their pain was triggered using a predetermined individualized thermal stimulus, which is characteristic of IEM.^14^ The primary end point (pain relief during the 0–4 hours period after dosing) was not met—presumably because the necessary time of maximum concentration was not reached, unlike data from healthy volunteers in phase I studies. However, a statistically significant difference was observed at 8 to 9 h after dosing (*P* = 0.03). In an interesting link to the preclinical study, PF-05089771 only reduced heat-evoked pain in the 3 participants whose iPSC-SN displayed reduced warmth-evoked hyperexcitability when exposed to the drug.^14^

PF-05089771 was also assessed in a randomized, double-blind study in 135 individuals with diabetic peripheral neuropathy,^45^ which evaluated the efficacy of the drug on its own and with pregabalin (a common treatment for peripheral neuropathy) to reduce pain from baseline in the PF-05089771 arm of the study after 4 weeks. A trend in pain reduction was reported, but this change was not statistically significant compared with placebo. The reduction in pain score from pregabalin was larger than that of PF-05089771 and statistically significant compared with placebo; the study did not continue to the second phase to evaluate PF-05089771 as an add-on to pregabalin. Another phase II trial with a 235-participant, randomized, double-blind, double-dummy, parallel group, placebo-controlled study assessed the efficacy of increasing doses of PF-05089771 in postoperative dental pain.^43^ The compound did not show superiority over ibuprofen.

The mismatch between preclinical and clinical data emphasized the need for a detailed knowledge of the plasma protein-bound and free-drug fractions, the ability to cross the blood–brain barrier and blood–nerve barrier, and the extent of target occupancy, which are crucial to understanding and predicting the efficacy of new drugs.^47^ Arylsulfonamide Nav1.7 blockers, like PF-05089771, have limited CNS penetration.^45^ Because Nav1.7 is present at the central terminal of dorsal root ganglion neurons in the dorsal horn behind the blood–spinal cord barrier,^12^ the block of Nav1.7 in the dorsal horn might be necessary to effectively inhibit pain signaling. There remains some uncertainty around the exact level of Nav1.7 blockade necessary to achieve analgesia. Using dynamic clamp electrophysiology and iPSC-SN, a recent study suggested that approximately 50% blockade of Nav1.7 currents can reverse neuronal hyperexcitability to baseline levels.^4^ A similar conclusion has also been reached using empirical data from rodents and nonhuman primates to model desirable pharmacocinetic/pharmacodynamic in humans for an effective target engagement.^7^ Although reduced excitability of neurons in culture might be a biomarker for an effective drug candidate, care must be exercised in equating it with reducing pain in patients. However, several animal studies suggested that channel occupancy by this class of Nav1.7 blockers required for efficacy might be higher than 50% and suggested the compound needed to achieve a plasma concentration of up to 50 times the IC_50_ to achieve significant pain reduction.^31,55^ At these concentrations, the compounds could lose their selectivity and block other neuronal sodium channels. This challenge is likely related to a high percentage of plasma protein binding (>99%), high unbound clearance, and in vitro hepatotoxicity.^32,46^

### 3.3. Summary and conclusions

The clinical trials to date have not yet revealed the Nav1.7-blocking magic bullet for pain and have been hampered by mixed results. Although Vitroxigene is not Nav1.7-selective, it is well tolerated and could be useful if proven effective in the larger clinical trials. The new study by Kraus et al.^39^ using a new arylsulfonamide Nav1.7 blocker with higher free-drug fraction points to improved pharmacokinetic properties of this class of molecules and is more effective than PF-05089771 in blocking conduction in sensory C-fibers and response to noxious heat in a nonhuman primate model, indicating that newer compounds in this class might overcome some of the challenges that affected the performance of PF-05089771 in clinical trials. This also suggests that the inhibition of Nav1.7 in the peripheral compartment might still be effective for pain treatment if sufficient free drug is present. There are also ongoing attempts to engineer and optimize naturally occurring Nav channel–targeting peptides, venoms^15,36^ and toxins, like JNJ63955918 (which is a Protoxin-II derivative) or ceratoxin-1,^30,53^ or subtype-specific antibodies.^11,44^ With these investigations underway, the development of Nav1.7-targeting drugs still maintains the promise of yielding new, safe, effective, and nonaddictive treatments for pain.

## 4. Neuropeptides: tachykinin receptor 1 antagonists blocking substance P effects

### 4.1. Tachykinin receptors and their involvement in disease

The tachykinin family of peptides (eg, substance P) interact through 3 neurokinin receptors (NKRs) encoded by 3 *Tacr* genes. They are involved in several physiological and pathophysiological processes.^48,54^ Neurokinin 1 receptor (NK1R) (SP receptor) was cloned from a rat brain cDNA library by functional expression in Xenopus oocytes and cross-hybridization to bovine NK2R. It is generally suggested that these peptides are only released due to strong cell activation and that receptor antagonists can only act in a sensitized or pathological system.^54^ Therefore, tachykinin receptors have been of huge interest in the treatment of various disease conditions including chronic pain.

Substance P was first described as eliciting atropine resistant smooth muscle contraction and hypotension of the blood pressure. Substance P is widely distributed and can be found not only in the central and peripheral nervous system but also in nonneuronal cells, eg, mast cells and body fluids.^48^ Painful stimuli release SP from presynaptic terminals of nociceptors in the dorsal horn. Similarly, SP is released in the periphery by means of antidromic stimulation of direct activation, eg, by TRPV1 stimulation. Substance P binds neurokinin 1 receptor on postsynaptic neurons transmitting nociceptive signals to the brain. Troves of preclinical data with NK1R antagonists supported a key role for SP in chronic pain. Substance P does not activate an action potential in nociceptors per se but rather sensitizes nociceptors to other chemical, thermal, or mechanical stimuli inducing a left shift of the activation curve.

### 4.2. Clinical studies and challenges

Neuropeptides: tachykinin receptor 1 antagonists can be divided into 2 groups: peptide and nonpeptide antagonists. Due to negative side effects such as neurotoxicity, peptide antagonists are not employed in clinical practice.^48^ Up to date, 5 nonpeptide antagonists are on the market to treat nausea and vomiting in patients undergoing chemotherapy,^42^ but none of them is approved for pain therapy*.* Clinical trials of highly selective NK1R antagonists were simply unsuccessful.^23,54^ Apparently, it is rather CGRP that mediates pain sensation while SP seems to be secondary.^54^

Several reasons can explain the ineffectiveness of NK1 receptor antagonists: species-specific differences in affinities of human vs preclinical animal models. Because most of the preclinical data came from rodents, lower affinity to the human NK1R is one of the reasons of failure. Because of the species differences, higher doses have been used, which could have led to other mechanisms of action including blockade of calcium influx. In addition, when NK1R antagonists were used in preclinical studies in the 1990s, disease mechanisms and molecular interactions were not comprehensively understood and the predictive value of animal pain behavior overestimated.^34,54^ For example, data are just beginning to emerge on molecular interaction of antagonist and receptor (allosteric/orthosteric) and then inhibition vs activation in the presence of the agonists and interactions between NKR and G protein–coupled receptor (GPCR, heterodimers). Furthermore, it seems that there is an inherent redundancy of the tachykinin system, allowing for escape mechanisms.^54^ Recent evidence points toward an important role of the appropriate subcellular domain, eg, endosomal signaling.^38,43^ Because many GPCR-targeted drugs are not cell permeable, antagonists would block GPCR function at the cell surface but not within endosomes.

### 4.3. Summary and conclusions

Although substance P can sensitize nociceptors in vitro and in vivo, genetic ablation preclinical studies suggested that it might not play a major role in multiple pain modalities. These data should have raised some concern during the drug development process.^20^ Furthermore, our inadequate understanding of the localization of the NKR1 in subcellular organelles and how to target them could improve chances of future NK1R antagonists to succeed in clinical trials. Furthermore, unexpected side effects have been reported with current NK1R antagonists because most of them are metabolized through CYP3A4 and thus contribute to potential drug–drug interactions.^51^ So, NK1R antagonists might be better used to treat nausea and vomiting associated with a variety of conditions. A completely new field is the use of NK1R in the treatment of pruritus—based on observations in animal models—so new trials are on the way (Clinical Trials NCT03836001).

## 5. Discussion: lessons learned

**Figure 1.**
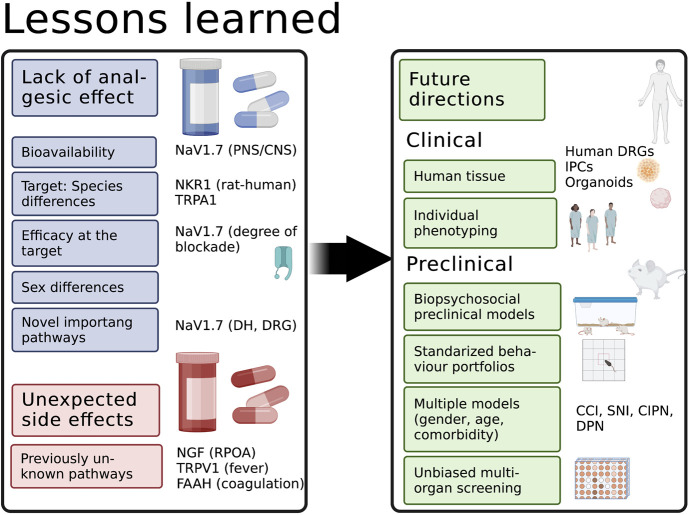
Summary of reasons for failure for selected pathways and possible future direction of pain research to foster translation of pain knowledge into practice. Failure of analgesics in clinical trials can be attributed to 2 categories: lack of effect and unwanted side effects. Examples are given in the right column and explained in the main text. Many suggestions for the future have been made based on these observations—mainly to address the lack of effect. CCI, chronic constriction injury; CIPN, chemotherapy-induced neuropathy; CNS, central nervous system; DH, dorsal horn; DPN, diabetic polyneuropathy; DRG, dorsal root ganglion; FAAH, fatty acid amide hydrolase; IPSC, induced pluripotent stem cells; NaV1.7, voltage-gated sodium channel; NGF, nerve growth factor; RPOA, rapid progressing osteoarthritis; SNI, spared nerve injury; TRPA1, transient receptor potential ankyrin 1; TRPV1, transient receptor potential vanilloid 1.

When targeting a well-established pathophysiological mechanism fails to lead to new treatment, several lessons can be learnt. Failures to translate preclinical data can be caused by the lack of sufficient efficacy and severe side effects (Fig. 1). Here, we provided evidence for the former exemplified by targeting NK1R and NaV1.7 or severe side effects as seen for NGF antibodies. Other examples for the former also include TRPV1 and transient receptor potential ankyrin 1 (TRPA1) antagonists. Fatty acid amide hydrolase blockers like 3-(1-(cyclohexyl(methyl)carbamoyl)-1H-imidazol-4-yl)pyridine 1-oxide to prevent the degradation of endocannabinoids failed due to unexpected death in phase I clinical trials probably due to off target effects on the inhibition of other lipases.^28^

To enhance the success of translation, future studies should always include human tissue or human nociceptors from iPSCs to assess species-specific effects on the target. Patients should be phenotyped in more detail to make sure that only those who are treated with a drug that is specific to the target in this pathway—aiming toward an individual pathophysiology possibly also across different disease entities. Last but not least, measuring nociception as well as pain will be a challenge for the future in the treatment of chronic pain in its biopsychosocial context. Funding agencies should keep these aspects in mind when evaluating grants and their possible translation in the future.

Importantly, we need more specific biomarkers allowing to identify patients who could best benefit from a specific treatment. Postmarket monitoring by the appropriate institutions and examination of class effects are necessary steps in this direction. Patients and clinicians can support this process by reporting unexpected symptoms or diseases to these surveillance agencies.

## Disclosures

H.L. Rittner recieved financial compensation from Algiax Pharmaceuticals GmbH, Grünenthal GmbH and Orion for consulting services.

## References

[1] Abdiche YN Malashock DS Pons J. Probing the binding mechanism and affinity of tanezumab, a recombinant humanized anti-NGF monoclonal antibody, using a repertoire of biosensors. Protein Sci 2008;17:1326–35. 18505735 10.1110/ps.035402.108 PMC2492818

[2] Alexandrou AJ Brown AR Chapman ML Estacion M Turner J Mis MA Wilbrey A Payne EC Gutteridge A Cox PJ Doyle R Printzenhoff D Lin Z Marron BE West C Swain NA Storer RI Stupple PA Castle NA Hounshell JA Rivara M Randall A Dib-Hajj SD Krafte D Waxman SG Patel MK Butt RP Stevens EB. Subtype-selective small molecule inhibitors reveal a fundamental role for Nav1.7 in nociceptor electrogenesis, axonal conduction and presynaptic release. PLoS One 2016;11:e0152405. 27050761 10.1371/journal.pone.0152405 PMC4822888

[3] Alsaloum M Higerd GP Effraim PR Waxman SG. Status of peripheral sodium channel blockers for non-addictive pain treatment. Nat Rev Neurol 2020;16:689–705. 33110213 10.1038/s41582-020-00415-2

[4] Alsaloum M Labau JIR Liu S Effraim P Waxman SG. Stem cell-derived sensory neurons modelling inherited erythromelalgia: normalization of excitability. Brain 2022;146:359–71. 10.1093/brain/awac031 PMC10060693 35088838

[5] A Phase IIa Withdrawal Study of CNV1014802 in Patients With Trigeminal Neuralgia, Vol. 2023. https://clinicaltrials.gov/ct2/show/NCT01540630. Accessed October 3, 2023.

[6] A randomized, double blind, cross-over study to evaluate the safety and efficacy of CNV1014802 in subjects with neuropathic pain from lumbosacral radiculopathy, Vol. 2023. https://www.clinicaltrialsregister.eu/ctr- search/trial/2010-023962-39/results. Accessed October 3, 2023.

[7] Ballard JE Pall PS Vardigan J Zhao F Holahan MA Zhou X Jochnowitz N Kraus RL Klein RM Henze DA Houghton AK Burgey CS Gibson C Struyk A. Translational pharmacokinetic-pharmacodynamic modeling of NaV1.7 inhibitor MK-2075 to inform human efficacious dose. Front Pharmacol 2021;12:786078. 35002718 10.3389/fphar.2021.786078 PMC8740778

[8] Baron R Dickenson AH Calvo M Dib-Hajj SD Bennett DL. Maximizing treatment efficacy through patient stratification in neuropathic pain trials. Nat Rev Neurol 2023;19:53–64. 36400867 10.1038/s41582-022-00741-7

[9] Bennett DL Clark AJ Huang J Waxman SG Dib-Hajj SD. The role of voltage-gated sodium channels in pain signaling. Physiol Rev 2019;99:1079–151. 30672368 10.1152/physrev.00052.2017

[10] Biogen Inc (BIIB) Q3 2018 earnings conference call transcript, 2018. Available at: https://www.fool.com/earnings/call-transcripts/2018/10/23/biogen-inc-biib-q3-2018-earnings-conference-call-t.aspx. Accessed October 3, 2023

[11] Biswas K Nixey TE Murray JK Falsey JR Yin L Liu H Gingras J Hall BE Herberich B Holder JR Li H Ligutti J Lin MHJ Liu D Soriano BD Soto M Tran L Tegley CM Zou A Gunasekaran K Moyer BD Doherty L Miranda LP. Engineering antibody reactivity for efficient derivatization to generate Na(V)1.7 inhibitory GpTx-1 peptide-antibody conjugates. ACS Chem Biol 2017;12:2427–35. 28800217 10.1021/acschembio.7b00542

[12] Black JA Frézel N Dib-Hajj SD Waxman SG. Expression of Nav1.7 in DRG neurons extends from peripheral terminals in the skin to central preterminal branches and terminals in the dorsal horn. Mol Pain 2012;8:82. 23134641 10.1186/1744-8069-8-82 PMC3517774

[13] Bueker ED. Implantation of tumors in the hind limb field of the embryonic chick and the developmental response of the lumbosacral nervous system. Anatomical Rec 1948;102:369–89. 10.1002/ar.1091020309 18098427

[14] Cao L McDonnell A Nitzsche A Alexandrou AJ Saintot P Loucif AJ Brown AR Young GT Mis MA Randall A Waxman SG Stanley P Kirby S Tarabar S Gutteridge A Butt RP Mckernan RM Whiting PJ Ali Z Bilsland JG Stevens EB. Pharmacological reversal of a pain phenotype in iPSC-derived sensory neurons and patients with inherited erythromelalgia. Sci Transl Med 2016;8:335ra56. 10.1126/scitranslmed.aad7653 27099175

[15] Cardoso FC Lewis RJ. Structure-function and therapeutic potential of spider venom-derived cysteine knot peptides targeting sodium channels. Front Pharmacol 2019;10:366. 31031623 10.3389/fphar.2019.00366 PMC6470632

[16] Caterina MJ Schumacher MA Tominaga M Rosen TA Levine JD Julius D. The capsaicin receptor: a heat-activated ion channel in the pain pathway. Nature 1997;389:816–24. 9349813 10.1038/39807

[17] Chen C-C Akopian AN Sivilottit L Colquhoun D Burnstock G Wood JN. A P2X purinoceptor expressed by a subset of sensory neurons. Nature 1995;377:428–31. 7566119 10.1038/377428a0

[18] Coste B Mathur J Schmidt M Earley TJ Ranade S Petrus MJ Dubin AE Patapoutian A. Piezo1 and Piezo2 are essential components of distinct mechanically activated cation channels. Science 2010;330:55–60. 20813920 10.1126/science.1193270 PMC3062430

[19] Crossover Study of CNV1014802 in Subjects With Neuropathic Pain From Lumbosacral Radiculopathy, Vol. 2023. https://clinicaltrials.gov/show/NCT01561027. Accessed October 3, 2023.

[20] De Felipe CD Herrero JF O'Brien JA Palmer JA Doyle CA Smith AJ Laird JM Belmonte C Cervero F Hunt SP. Altered nociception, analgesia and aggression in mice lacking the receptor for substance P. Nature 1998;392:394–7. 9537323 10.1038/32904

[21] Deen M Correnti E Kamm K Kelderman T Papetti L Rubio-Beltrán E Vigneri S Edvinsson L Maassen Van Den Brink A. Blocking CGRP in migraine patients—a review of pros and cons. J Headache Pain 2017;18:96. 28948500 10.1186/s10194-017-0807-1 PMC5612904

[22] Deuis JR Wingerd JS Winter Z Durek T Dekan Z Sousa SR Zimmermann K Hoffmann T Weidner C Nassar MA Alewood PF Lewis RJ Vetter I. Analgesic effects of GpTx-1, PF-04856264 and CNV1014802 in a mouse model of NaV1.7-mediated pain. Toxins (Basel) 2016;8:78. 26999206 10.3390/toxins8030078 PMC4810223

[23] Di Fabio R Alvaro G Braggio S Carletti R Gerrard PA Griffante C Marchioro C Pozzan A Melotto S Poffe A Piccoli L Ratti E Tranquillini E Trower M Spada S Corsi M. Identification, biological characterization and pharmacophoric analysis of a new potent and selective NK1 receptor antagonist clinical candidate. Bioorg Med Chem 2013;21:6264–73. 24075145 10.1016/j.bmc.2013.09.001

[24] Dib-Hajj SD Cummins TR Black JA Waxman SG. Sodium channels in normal and pathological pain. Annu Rev Neurosci 2010;33:325–47. 20367448 10.1146/annurev-neuro-060909-153234

[25] Dib-Hajj SD Yang Y Black JA Waxman SG. The Na(V)1.7 sodium channel: from molecule to man. Nat Rev Neurosci 2013;14:49–62. 23232607 10.1038/nrn3404

[26] Efficacy and Safety Study of BIIB074 in Participants With Small Fiber Neuropathy, Vol. 2023. https://clinicaltrials.gov/ct2/show/results/NCT03339336. Accessed October 3, 2023.

[27] Enomoto M Mantyh PW Murrell J Innes JF Lascelles BDX. Anti-nerve growth factor monoclonal antibodies for the control of pain in dogs and cats. Vet Rec 2019;184:23. 30368458 10.1136/vr.104590 PMC6326241

[28] Feldwisch-Drentrup H. New clues to why a French drug trial went horribly wrong Study suggests a candidate drug had effects on a range of enzymes involved in the brain’s metabolism, 2017, Vol. 2023. https://www.science.org/content/article/new-clues-why-french-drug-trial-went-horribly-wrong. Accessed October 3, 2023

[29] Finnerup NB Attal N Haroutounian S McNicol E Baron R Dworkin RH Gilron I Haanpää M Hansson P Jensen TS Kamerman PR Lund K Moore A Raja SN Rice AS Rowbotham M Sena E Siddall P Smith BH Wallace M. Pharmacotherapy for neuropathic pain in adults: a systematic review and meta-analysis. Lancet Neurol 2015;14:162–73. 25575710 10.1016/S1474-4422(14)70251-0 PMC4493167

[30] Flinspach M Xu Q Piekarz AD Fellows R Hagan R Gibbs A Liu Y Neff RA Freedman J Eckert WA Zhou M Bonesteel R Pennington MW Eddinger KA Yaksh TL Hunter M Swanson RV Wickenden AD. Insensitivity to pain induced by a potent selective closed-state Nav1.7 inhibitor. Sci Rep 2017;7:39662. 28045073 10.1038/srep39662 PMC5206724

[31] Focken T Chowdhury S Zenova A Grimwood ME Chabot C Sheng T Hemeon I Decker SM Wilson M Bichler P Jia Q Sun S Young C Lin S Goodchild SJ Shuart NG Chang E Xie Z Li B Khakh K Bankar G Waldbrook M Kwan R Nelkenbrecher K Karimi Tari P Chahal N Sojo L Robinette CL White AD Chen CA Zhang Y Pang J Chang JH Hackos DH Johnson JP Jr Cohen CJ Ortwine DF Sutherlin DP Dehnhardt CM Safina BS. Design of conformationally constrained acyl sulfonamide isosteres: identification of N-([1,2,4]Triazolo[4,3- a]pyridin-3-yl)methane-sulfonamides as potent and selective hNa(V)1.7 inhibitors for the treatment of pain. J Med Chem 2018;61:4810–31. 29737846 10.1021/acs.jmedchem.7b01826

[32] Graceffa RF Boezio AA Able J Altmann S Berry LM Boezio C Butler JR Chu-Moyer M Cooke M DiMauro EF Dineen TA Feric Bojic E Foti RS Fremeau RT Jr Guzman-Perez A Gao H Gunaydin H Huang H Huang L Ilch C Jarosh M Kornecook T Kreiman CR La DS Ligutti J Milgram BC Lin MHJ Marx IE Nguyen HN Peterson EA Rescourio G Roberts J Schenkel L Shimanovich R Sparling BA Stellwagen J Taborn K Vaida KR Wang J Yeoman J Yu V Zhu D Moyer BD Weiss MM. Sulfonamides as selective Na(V)1.7 inhibitors: optimizing potency, pharmacokinetics, and metabolic properties to obtain atropisomeric quinolinone (AM-0466) that affords robust in vivo activity. J Med Chem 2017;60:5990–6017. 28324649 10.1021/acs.jmedchem.6b01850

[33] Harrison L. New sodium channel blocker dampens radiculopathy pain. Medscape Medical News, 2015. Available at: https://www.medscape.com/viewarticle/845522. Accessed October 3, 2023.

[34] Hill R. NK1 (substance P) receptor antagonists–why are they not analgesic in humans? Trends Pharmacol Sci 2000;21:244–6. 10871891 10.1016/s0165-6147(00)01502-9

[35] Hinckley CA Kuryshev YA Sers A Barre A Buisson B Naik H Hajós M. Characterization of vixotrigine, a broad-spectrum voltage-gated sodium channel blocker. Mol Pharmacol 2021;99:49–59. 33298520 10.1124/molpharm.120.000079

[36] Israel MR Tay B Deuis JR Vetter I. Sodium channels and venom peptide pharmacology. Adv Pharmacol 2017;79:67–116. 28528674 10.1016/bs.apha.2017.01.004

[37] Jankowski MP Koerber HR. Frontiers in neuroscience. In: Kruger L Light AR, editors. Translational pain research: from mouse to man. Boca Raton, FL: Taylor and Francis Group, LLC, 2010. 21882466

[38] Jensen DD Lieu T Halls ML Veldhuis NA Imlach WL Mai QN Poole DP Quach T Aurelio L Conner J Herenbrink CK Barlow N Simpson JS Scanlon MJ Graham B McCluskey A Robinson PJ Escriou V Nassini R Materazzi S Geppetti P Hicks GA Christie MJ Porter CJH Canals M Bunnett NW. Neurokinin 1 receptor signaling in endosomes mediates sustained nociception and is a viable therapeutic target for prolonged pain relief. Sci Transl Med 2017;9:eaal3447. 28566424 10.1126/scitranslmed.aal3447 PMC6034632

[39] Kraus RL Zhao F Pall PS Zhou D Vardigan JD Danziger A Li Y Daley C Ballard JE Clements MK Klein RM Holahan MA Greshock TJ Kim RM Layton ME Burgey CS Serra J Henze DA Houghton AK. Nav1.7 target modulation and efficacy can be measured in nonhuman primate assays. Sci Transl Med 2021;13:eaay1050. 34011626 10.1126/scitranslmed.aay1050

[40] Kumar V Mahal BA. Ngf—the TrkA to successful pain treatment. J Pain Res 2012;5:279–87. 23028238 10.2147/JPR.S33408 PMC3442742

[41] Lane NE Schnitzer TJ Birbara CA Mokhtarani M Shelton DL Smith MD Brown MT. Tanezumab for the treatment of pain from osteoarthritis of the knee. N Engl J Med 2010;363:1521–31. 20942668 10.1056/NEJMoa0901510 PMC6896791

[42] Lanzarotti C Rossi G. Effect of netupitant, a highly selective NK1 receptor antagonist, on the pharmacokinetics of midazolam, erythromycin, and dexamethasone. Support Care Cancer 2013;21:2783–91. 23729226 10.1007/s00520-013-1855-y

[43] Latorre R Ramírez-Garcia PD Hegron A Grace JL Retamal JS Shenoy P Tran M Aurelio L Flynn B Poole DP Klein-Cloud R Jensen DD Davis TP Schmidt BL Quinn JF Whittaker MR Veldhuis NA Bunnett NW. Sustained endosomal release of a neurokinin-1 receptor antagonist from nanostars provides long-lasting relief of chronic pain. Biomaterials 2022;285:121536. 35533442 10.1016/j.biomaterials.2022.121536 PMC10064865

[44] Liu D Tseng M Epstein L Green L Chan B Soriano B Lim D Pan O Murawsky C King C Moyer B. Evaluation of recombinant monoclonal antibody SVmab1 binding to NaV1.7 target sequences and block of human NaV1.7 currents [version 1; peer review: 3 approved]. F1000Research 2016;5:2764. 27990272 10.12688/f1000research.9918.1 PMC5155501

[45] McDonnell A Collins S Ali Z Iavarone L Surujbally R Kirby S Butt RP. Efficacy of the Nav1.7 blocker PF-05089771 in a randomised, placebo-controlled, double-blind clinical study in subjects with painful diabetic peripheral neuropathy. PAIN 2018;159:1465–76. 29578944 10.1097/j.pain.0000000000001227

[46] McKerrall SJ Nguyen T Lai KW Bergeron P Deng L DiPasquale A Chang JH Chen J Chernov-Rogan T Hackos DH Maher J Ortwine DF Pang J Payandeh J Proctor WR Shields SD Vogt J Ji P Liu W Ballini E Schumann L Tarozzo G Bankar G Chowdhury S Hasan A Johnson JP Khakh K Lin S Cohen CJ Dehnhardt CM Safina BS Sutherlin DP. Structure- and ligand-based discovery of chromane arylsulfonamide Na_v_1.7 inhibitors for the treatment of chronic pain. J Med Chem 2019;62:4091–109. 30943032 10.1021/acs.jmedchem.9b00141

[47] Mulcahy JV Pajouhesh H Beckley JT Delwig A Du Bois J Hunter JC. Challenges and opportunities for therapeutics targeting the voltage-gated sodium channel isoform Na_V_1.7. J Med Chem 2019;62:8695–710. 31012583 10.1021/acs.jmedchem.8b01906 PMC6786914

[48] Muñoz M Coveñas R. Neurokinin receptor antagonism: a patent review (2014-present). Expert Opin Ther Patents 2020;30:527–39. 10.1080/13543776.2020.1769599 32401556

[49] Schaible HG. The fine line between innovation and risk: is anti-NGF a pain medication of the future? Schmerz 2010;24:559–60. 21057818 10.1007/s00482-010-0993-0

[50] Schaible HG. Pain therapy using anti-nerve growth factor antibodies?: pain research in a dilemma. Schmerz 2021;35:301–3. 34542707 10.1007/s00482-021-00574-1

[51] Schoffelen R Lankheet AG van Herpen CML van der Hoeven JJM Desar IME Kramers C. Drug-drug interactions with aprepitant in antiemetic prophylaxis for chemotherapy. Neth J Med 2018;76:109–14. 29667586

[52] Sexton JE Cox JJ Zhao J Wood JN. The genetics of pain: implications for therapeutics. Annu Rev Pharmacol Toxicol 2018;58:123–42. 28968191 10.1146/annurev-pharmtox-010617-052554

[53] Shcherbatko A Rossi A Foletti DL Zhu G Bogin O Galindo Casas M Rickert M Hasa-Moreno A Bartsevich VV Crameri A Steiner AR Henningsen RA Gill A Pons J Shelton DL Rajpal A Strop P. Engineering highly potent and selective microproteins against Nav1.7 sodium channel for treatment of pain. J Biol Chem 2016;291:13974–86. 27129258 10.1074/jbc.M116.725978 PMC4933158

[54] Steinhoff MS von Mentzer B Geppetti P Pothoulakis C Bunnett NW. Tachykinins and their receptors: contributions to physiological control and the mechanisms of disease. Physiol Rev 2014;94:265–301. 24382888 10.1152/physrev.00031.2013 PMC3929113

[55] Storer RI Pike A Swain NA Alexandrou AJ Bechle BM Blakemore DC Brown AD Castle NA Corbett MS Flanagan NJ Fengas D Johnson MS Jones LH Marron BE Payne CE Printzenhoff D Rawson DJ Rose CR Ryckmans T Sun J Theile JW Torella R Tseng E Warmus JS. Highly potent and selective Na_V_1.7 inhibitors for use as intravenous agents and chemical probes. Bioorg Med Chem Lett 2017;27:4805–11. 29029933 10.1016/j.bmcl.2017.09.056

[56] Study to Evaluate the Efficacy and Safety of BIIB074 in Neuropathic Pain From Lumbosacral Radiculopathy (RELAY-1), Vol. 2023. https://clinicaltrials.gov/show/NCT02935608. Accessed October 3, 2023.

[57] Theodosiou M Rush AR Zhou FX Hu D Walker SJ Tracey JD. Hyperalgesia due to nerve damage: role of nerve growth factor. PAIN 1999;81:245–55. 10431712 10.1016/S0304-3959(99)00018-4

[58] Wise BL Seidel MF Lane NE. The evolution of nerve growth factor inhibition in clinical medicine. Nat Rev Rheumatol 2021;17:34–46. 33219344 10.1038/s41584-020-00528-4

[59] Zakrzewska JM Palmer J Morisset V Giblin GMP Obermann M Ettlin DA Cruccu G Bendtsen L Estacion M Derjean D Waxman SG Layton G Gunn K Tate S. Safety and efficacy of a Nav1.7 selective sodium channel blocker in patients with trigeminal neuralgia: a double-blind, placebo-controlled, randomised withdrawal phase 2a trial. Lancet Neurol 2017;16:291–300. 28216232 10.1016/S1474-4422(17)30005-4

